# Enhancing Stability of Metallic Magnesium Nanoparticles toward Oxidation in Water via PEG‐Phosphonate Passivation

**DOI:** 10.1002/chem.202503619

**Published:** 2026-04-11

**Authors:** Anupong Nuekaew, Delphine Talbot, Ali Abou‐Hassan

**Affiliations:** ^1^ PHysicochimie des Électrolytes et Nanosystèmes InterfaciauX (PHENIX) CNRS, Sorbonne Université Paris F‐75005 France; ^2^ Institut Universitaire de France (IUF) Paris 75231 Cedex 05 France

**Keywords:** colloidal synthesis, magnesium nanoparticles, oxidation resistance, phosphonic acid PEG ligands, surface functionalization

## Abstract

Metallic magnesium nanoparticles (MgNPs) offer unique opportunities for nanoplasmonics due to their optical properties, sustainability, and low cost. Yet their rapid oxidation in water severely limits practical use. In this work, we report the structural stabilization of colloidally synthesized MgNPs toward oxidation through surface functionalization with α‐methoxy‐ω‐phosphonic acid poly(ethylene glycol) (PPEG1000). Three functionalization strategies were evaluated: post‐synthesis grafting, one‐pot addition after nucleation, and pre‐addition of PPEG prior to MgNP formation. The role of functionalization timing on nanoparticle morphology and structural stability was assessed. TEM analysis shows that bare MgNPs form well‐defined hexagonal platelets, whereas early PPEG addition disrupts Mg(II) reduction, yielding polymer‐embedded aggregates. In contrast, one‐pot introduction after initial nucleation arrests further growth, producing a star‐like morphology, while post‐functionalization retains the anisotropic platelet morphology. FTIR and TGA confirm phosphonate binding and PPEG surface coverage, with polymer loadings of 18 wt% (one‐pot) and 4 wt% (post‐functionalized). Water‐dispersion assays reveal dramatically improved stability, extending from minutes for bare MgNPs to 40 min (one‐pot) and 3 h (post‐functionalized). These findings establish phosphonic‐acid PEG ligands as effective passivating agents for MgNPs as a key parameter for morphology and stability control in aqueous environments.

## Graphical abstract

Metallic magnesium nanoparticles are stabilized against rapid oxidation in water through surface passivation with phosphonic acid–terminated poly(ethylene glycol). By controlling the timing of ligand addition during synthesis, nanoparticle growth, morphology, and aqueous lifetime are tuned, extending stability from minutes to hours while preserving plasmonic properties.

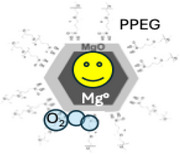

## 1 Introduction

In recent years, metallic magnesium nanoparticles (MgNPs) have gained considerable attention due to their unique plasmonic properties, sustainability, low cost, and biocompatibility. In contrast to NPs made from noble metals, MgNPs exhibit plasmonic resonances spanning the UV–Vis–NIR regions, due to their low optical losses across this range [1]. Early wet‐chemical routes were pioneered by Rieke and Bales, who reported the reduction of magnesium halides with potassium in tetrahydrofuran (THF) to produce activated magnesium powders [2]. Later, Rieke et al. introduced naphthalene as an electron carrier, improving the efficiency of the process [3]. More recently, MgNPs have been obtained via reduction of di‐n‐butylmagnesium with lithium naphthalenide, yielding diverse morphologies such as hexagonal platelets, rod‐like, and twinned structures [4]. In addition, it has been reported that MgNPs spontaneously form a thin self‐limiting oxide layer that passivates the metallic core, which can be stable in air for weeks [1, 4, 5, 6]. Several reaction parameters have been optimized, providing more control over NP size with a narrow size distribution [7]. However, in aqueous media, MgNPs oxidize rapidly, typically within 5 min, forming magnesium hydroxide and hydrogen gas. This instability severely restricts their exploitation required in aqueous media, including plasmonic and potential biomedical applications.

Surface functionalization strategies that can inhibit oxidation and improve dispersibility are therefore essential. One of the approaches used in literature on bulk magnesium and magnesium alloys is to employ polymers with a binding head group, such as a carboxylate or phosphonate ion with an aliphatic tail. Particularly, phosphonic acids have been extensively used to form robust self‐assembled monolayers (SAMs) via mono‐, bi‐, or tridentate binding to bulk surface oxides and hydroxides, using both liquid‐ and vapor‐phase deposition methods [8, 9]. Longer‐chain phosphonic acids, such as octadecylphosphonic acid, provided enhanced chemical stability in aqueous environments and improved corrosion resistance with hydrophobic properties [9]. Spectroscopic analyses (XPS, FTIR, AFM) confirmed the formation of stable phosphonate‐Mg/MgO bonding over extended periods, rendering this approach attractive to be used as a strategy at the nanoscale. Literature reports agree that phosphonic acid as a head group provides higher binding affinity and superior anticorrosive performance as compared to carboxylic acid [10, 11].

Furthermore, phosphonic acid functionalization can act as a surface‐level dopant, tuning the reactivity and selectivity of metal oxides. For example, modification of metal oxides with phosphonic acid monolayers alters catalytic pathways, such as alcohol dehydration, by controlling the near‐surface environment [12]. These findings indicate that phosphonic acid functionalization can influence surface properties beyond simple protection, offering additional functionality at the nanoscale. The combination of a phosphonic acid anchor with a PEG chain offers a dual‐function passivation strategy for stabilizing MgNPs. While the phosphonate group ensures strong binding to the Mg/MgO surface, the PEG segment introduces a highly hydrated, flexible corona that promotes dispersibility in water, suppresses aggregation, and provides a steric barrier against rapid oxidation [13]. PEG is also widely recognized for its biocompatibility and resistance to nonspecific adsorption, making it well‐suited for MgNPs intended for aqueous or bio‐relevant applications.

Here, we report the functionalization of colloidally synthesized MgNPs with α‐methoxy‐ω‐phosphonic acid poly(ethylene glycol) (PPEG1000). The phosphonate moiety is expected to anchor strongly to the Mg/MgO surface, while the PEG chain provides hydrophilicity and dispersibility in water. To investigate the effect of functionalization timing, we explored two strategies: (i) one‐pot synthesis, where PPEG was introduced during MgNP formation, and (ii) post‐synthesis functionalization, where preformed MgNPs were subsequently treated with PPEG. These approaches aim to stabilize MgNPs in aqueous media, retain their plasmonic properties, and provide a foundation for further studies on surface‐tuned functionality in nanoscale Mg‐based systems.

## 2 Results and Discussion

**FIGURE 1:**
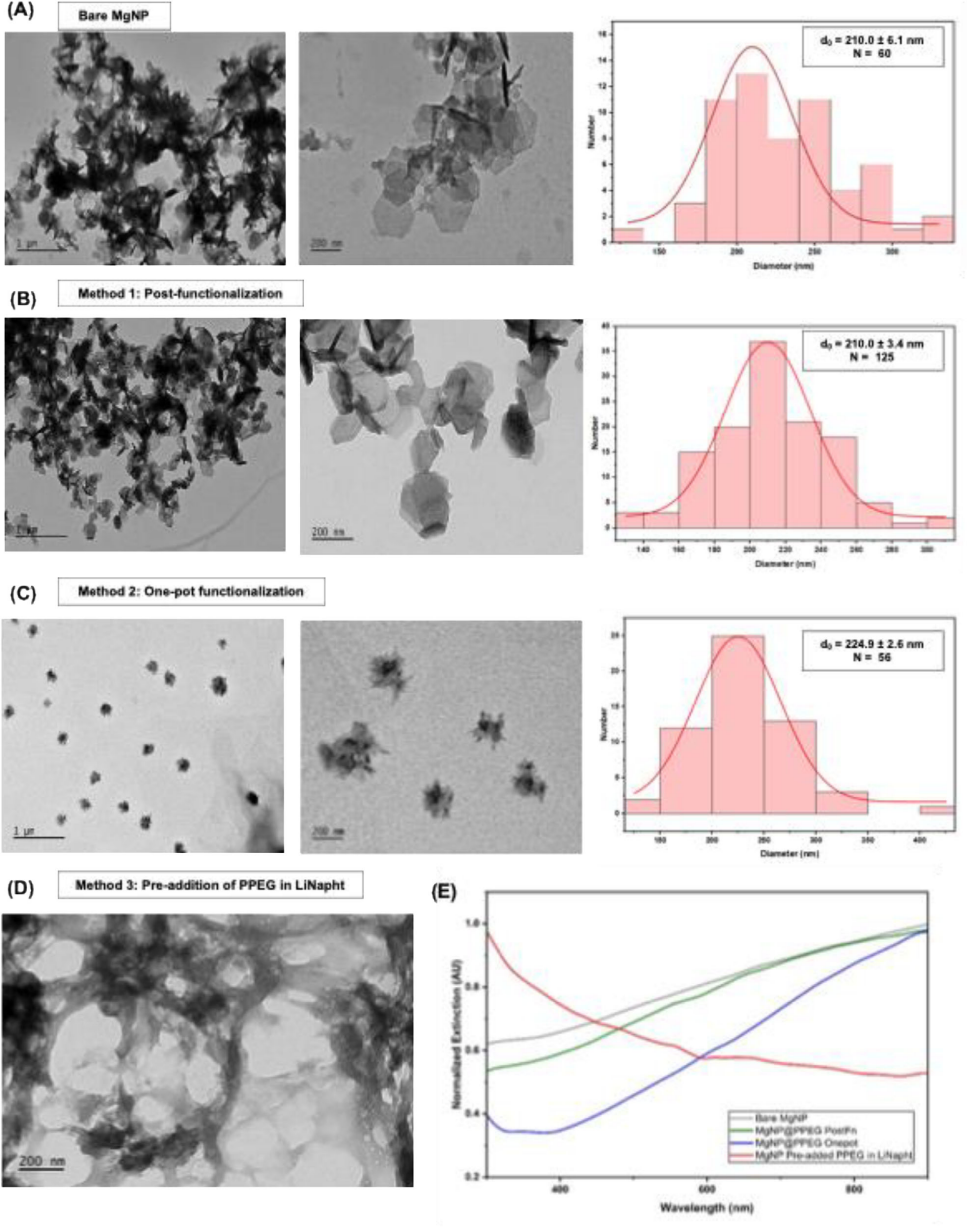
TEM images of MgNPs synthesized according to the different methods and their corresponding size distribution histograms. Four samples are shown: (A) bare MgNPs, (B) MgNP‐PPEG obtained by post‐functionalization (method 1), (C) MgNP‐PPEG obtained by one‐pot synthesis (method 2), and (D) MgNP‐PPEG synthesized in the presence of PPEG pre‐added in LiNapht (method 3). The histograms display the particle size distributions obtained from TEM image analysis, showing the effect of the different functionalization strategies on the size and shape of the MgNPs. (E) Normalized UV‐Vis‐NIR spectra of the final suspensions in IPA obtained using the different methods.

As a reference, bare MgNPs were synthesized through the reduction reaction of the precursor di‐n‐butylmagnesium by lithium naphthalenide (LiNapht) as a reducing agent in THF as described in the literature [14]. TEM images of the as‐synthesized bare MgNPs and the relative size distribution histogram are presented in Figure 1A. They show that the NPs are hexagonal nanoplatelets with an average diameter of 210.0 ± 6.1 nm with a thickness of 23.9 ± 7.2 nm. Typically, MgNPs are spontaneously passivated by a self‐limiting oxide shell (≤10 nm) due to oxidation in air at room temperature. By taking advantage of this oxide layer and the strong bridging ability of phosphonic acid as an anchoring group toward metal oxide surfaces through its multiple binding modes (such as mono‐, bi‐, tridentate modes of P‐O‐M bonds), we then functionalized post‐synthetically (method 1) MgNPs with Poly(ethylene glycol), α‐methoxy, ω‐phosphonic acid (PPEG1000). PEG chains are expected to provide a barrier toward water, limiting the oxidation while providing a good colloidal stability. To avoid the aggregation of MgNPs during the introduction of the ligand and ensure uniform binding of PPEG, the suspension was sonicated in an ultrasonication bath. The TEM image and the size distribution histograms in Figure 1B show that the structural integrity of MgNPs was preserved, as expected, after the post‐functionalization step. It should be noted that TEM samples were prepared by drop‐casting and solvent evaporation, which can induce aggregation artifacts. Hence, TEM images primarily provide information on particle size and morphology, while the colloidal state of the suspensions is assessed by UV–Vis–NIR spectroscopy.

Star‐like morphologies (Figure 1C) were observed for the one‐pot sample of MgNP@PPEG (method 2) when PPEG was injected after the magnesium precursor, resulting in MgNPs with an average diameter of 224.9 ± 2.6 nm. In this method, initially LiNapht was allowed to react with di‐n‐butylmagnesium briefly to initiate the nucleation of Mg^0^ and anisotropic growth. Upon PPEG addition, a portion of napthalenide anion gets protonated and consumed, lowering the effective reducing power of LiNapht, while phosphonate‐PEG coordinates to Mg species, arresting further crystalline growth. Thus, the hexagonal platelets cannot be fully developed, yielding clustered, star‐like aggregates.

TEM images of MgNP@PPEG obtained via pre‐addition of PPEG (method 3) revealed irregular dark regions with aggregated features (Figure 1D), suggesting poor Mg formation in a polymer matrix. The phenomenon is potentially caused by the incomplete reduction of di‐n‐butylmagnesium as the effective reducing strength of LiNapht decreases in the presence of PPEG. LiNapht is a very strong electron donor (*E*° ≈ −2.5 V vs. SHE) required for Mg(II) reduction (*E*° ≈ −2.37 V vs. SHE) and additionally acts as a strong base that can deprotonate the phosphonic acid in the reaction mixture, generating phosphonate anions. We therefore hypothesize that upon naphthalenide radical anion formation, its reducing ability was scavenged or diminished due to protonation (from phosphonic acid), converting Napht radical anion to naphthalene, resulting in inhibition or decrease of the nucleation of MgNPs. In parallel, the resulting phosphonate anions immediately coordinate Mg^2+^ upon the injection of Mg precursor, forming a stable Mg‐phosphonate complex. This leads to a slow formation of Mg^0^ nuclei as aggregates embedded into the polymeric phosphonate matrix.

The extinction spectra of all samples in IPA were acquired by UV‐Vis‐NIR spectroscopy (Figure 1E). The bare MgNPs exhibit a broad extinction profile with a gradual increase toward longer wavelengths, which can be attributed to particle aggregation in the absence of stabilizing agent, size and shape distribution [7]. The functionalization using method 1 resulted in the modest modification of the extinction profile as the extinction was slightly enhanced across the Vis‐NIR region compared to bare MgNPs. This trend suggests partial surface passivation, which improves colloidal stability. In contrast, MgNPs synthesized by method 2 exhibit a reduced extinction at a shorter wavelength and a smooth increase toward longer wavelength, showing more surface passivation and colloidal stability in IPA. Although TEM reveals star‐like morphologies, such anisotropy does not produce distinct optical features in the spectra, and the extinction response is dominated by aggregation and surface passivation effects. In contrast, the functionalization using method 1 showed a different extinction profile with the pronounced extinction at short wavelengths, which is the characteristic of strong light scattering from large aggregates, leading to poorly defined particle assemblies as pre‐addition of PPEG interferes with the controlled nucleation and growth. The color of bare MgNP and functionalization using method 1 was black and visually similar (Figure S2), whereas samples synthesized by using method 2 appeared blue. The sample obtained by method 3 showed a black color with a shade of yellowish brown.

**FIGURE 2:**
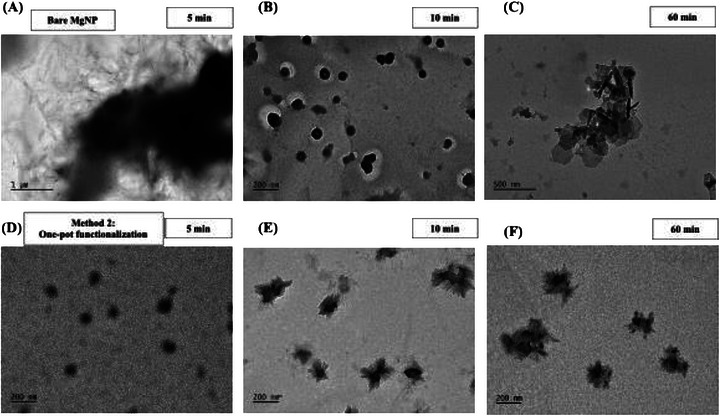
TEM images of MgNPs formed at different reaction times. Bare MgNPs at (A) 5 min, (B) 10 min, and (C) 60 min; MgNP‐PPEG samples synthesized by the one‐pot method (method 2) at (D) 5 min, (E) 10 min, and (F) 60 min. All other reaction parameters (reactant concentration, temperature = 53°C, stirring speed = 400 rpm) were kept constant.

The synthesis of bare MgNPs was tracked by varying the reaction time. At 5 min (Figure 2A), the particles were indistinguishable by TEM due to the early nucleation stage. Individual particles cannot be resolved, potentially due to high reactivity towards air and moisture in the absence of a stabilizing ligand during sample preparation. After 10 min (Figure 2B), the product consisted of small, quasi‐spherical nanoparticles forming small clusters or aggregates, suggesting an early stage of nucleation and growth. In contrast, after 60 min (Figure 2C), the particles evolved into well‐defined plate‐like structures, predominantly of a hexagonal shape. These particles show larger lateral dimensions and often appear stacked or overlapped, indicating the growth and evolution of shape after the rapid reduction of Mg^2+^ by LiNapht following the nucleation. It is noteworthy that LiNapht does not act as a surface‐active ligand. Based on FTIR analysis (Figure S2), no characteristic naphthalene bands were observed, suggesting that LiNapht is unlikely to associate with the nanoparticle surface after purification.

Concerning method 2, the reaction time was varied after adding PPEG from 5 to 60 min while keeping the reaction time between LiNapht and di‐n‐butylmagnesium constant (Figure 2E, F). In contrast to bare MgNPs, the one‐pot functionalization at 5 min (Figure 2D) showed more distinguishable and discrete nanoparticles, probably due to the presence of PPEG at the early nucleation stage. The particles at 10 and 60 min (Figure 2E, F) have a star‐like morphology with a modest increase in average size from 210.0 ± 3.0 to 224.9 ± 2.6 nm. This indicates that the NP growth occurs during the early stage of the reaction, while the growth becomes slower, resulting from surface passivation by PPEG while preserving the overall morphology.

Overall, our observations highlight that the timing of PPEG injection with regard to di‐n‐butylmagnesium addition is important to control MgNP synthesis. Upstream introduction of PPEG interferes with nucleation, yielding undefined morphologies, whereas downstream addition limits the growth and fixes the morphology into star‐like nanoclusters. Varying the delay time before PPEG addition could enable progressive shape control from irregular assemblies toward more well‐defined anisotropic platelets.

**FIGURE 3:**
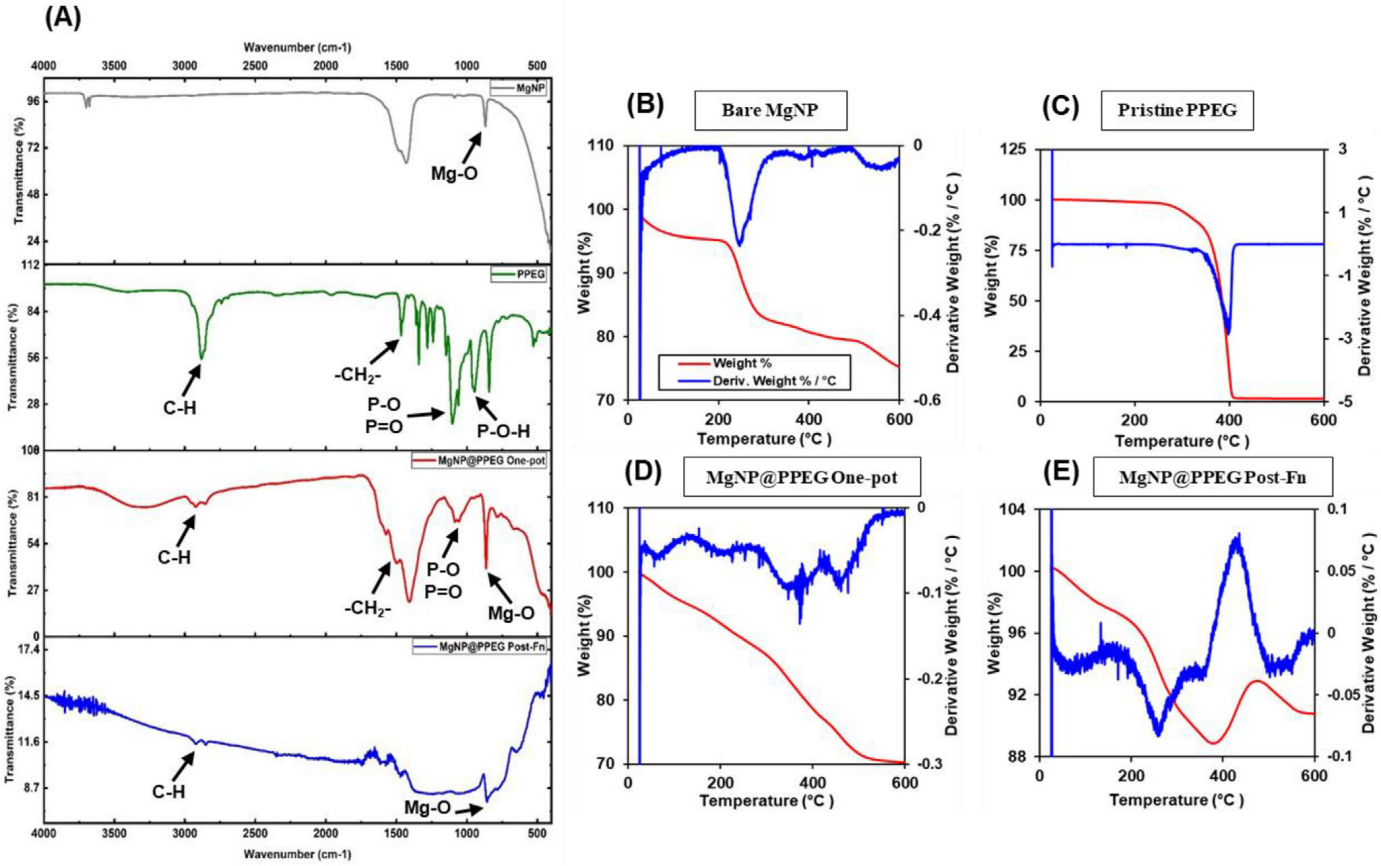
(A) FTIR spectra of bare MgNPs, pristine PPEG, MgNP‐PPEG obtained by one‐pot synthesis (method 2), and MgNP‐PPEG obtained by post‐functionalization (method 1). (B–E) Thermogravimetric analysis (TGA) curves of bare MgNPs, pristine PPEG, one‐pot MgNP‐PPEG (method 2), and post‐functionalized MgNP‐PPEG (method 1).

FTIR was used to assess the chemical composition and surface chemistry of all synthesized NPs (Figure 3A). All samples were oven‐dried prior to measurements. The dry powder bare MgNP appeared grey, indicating MgO was formed on the surface, whereas the post‐functionalized and one‐pot samples (methods 1 and 2) appeared black, indicating resistance toward air during the drying process.

**FIGURE 4:**
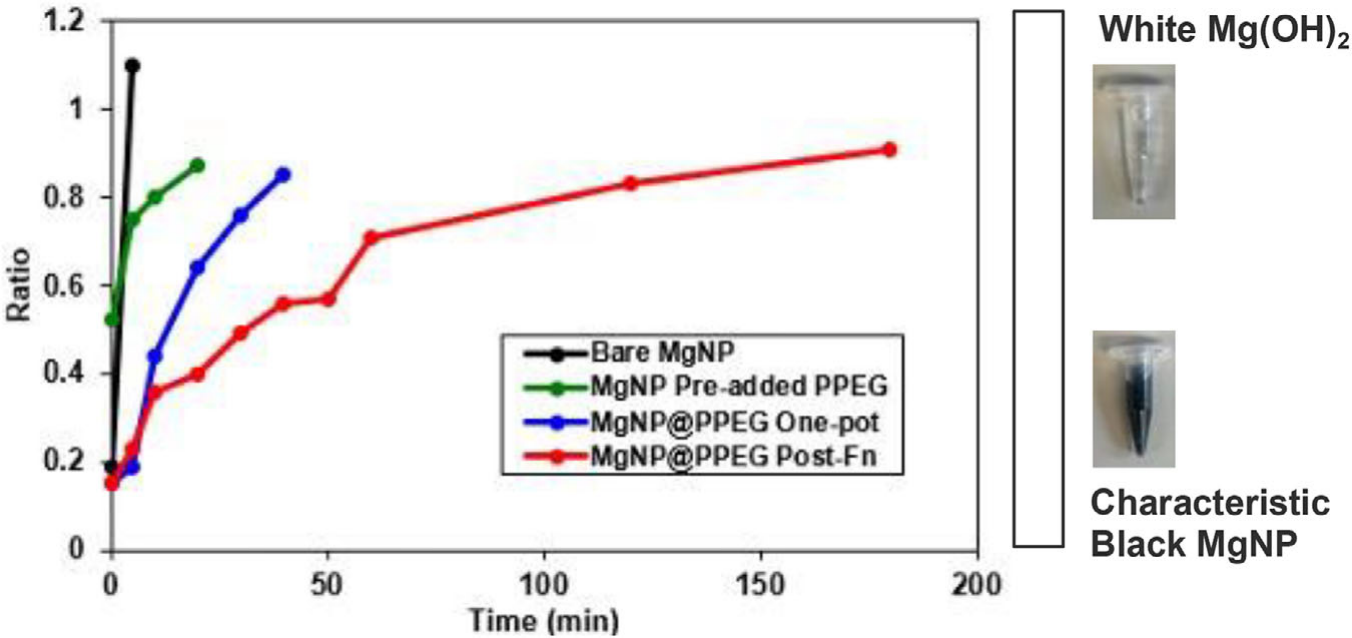
Colorimetric ratio versus time for bare MgNPs, MgNP‐PPEG synthesized with pre‐added PPEG in LiNapht, one‐pot MgNP‐PPEG, and post‐functionalized MgNP‐PPEG. The ratio was obtained by dividing the color intensity of a selected area by the intensity of the background, showing the color evolution of each sample and stability in aqueous media over time.

The FTIR spectrum of bare MgNPs and functionalized samples (methods 1 and 2) shows a characteristic band around ∼850 cm^−1^, corresponding to the Mg─O stretching vibration mode, which confirms the presence of a surface oxide layer [15]. The one‐pot sample (method 2) exhibits additional bands arising from the PPEG ligand. In particular, bands at ∼1496 and ∼2922 cm^−1^ are attributed to the C‐H stretching and bending modes of –CH_2_ groups within the PEG backbone. Strong bands in the 1000–1300 cm^−1^ region correspond to P─O and P═O stretching vibrations associated with the phosphonate group [16, 17]. Notably, the P─O─H band observed in pristine PPEG around 900–950 cm^−1^ is absent in the one‐pot sample (method 2), resulting from deprotonation of the phosphonic acid group and coordination to the Mg surface through P─O─Mg bonding [18]. This observation supports successful surface passivation of the nanoparticles by PEG‐phosphonate ligands. For the post‐functionalized sample, the coordination of PPEG on the surface could not be unambiguously resolved owing to the strong absorption of the hybrid material. Nonetheless, characteristic PPEG bands, including C─H stretching bands at ∼2851 and ∼2918 cm^−1^ remain visible, confirming the presence of the polymer on the Mg surface. Importantly, the absence of the naphthalene fingerprint region in all spectra ruled out the presence of naphthalene‐based by‐products or residual impurities after purification (Figure S2). The assignments of the main FTIR bands are summarized in Table S1.

Thermogravimetric analysis (TGA) under controlled N_2_ atmosphere was used as a complementary technique to FTIR to quantify the amount of the polymer. As a control, the TGA measurements of bare MgNPs (Figure 3B) and the pristine PPEG polymer (Figure 3C) were recorded first. For bare MgNPs (Figure 3B), the TGA showed a 20% mass loss until 250°C, probably due to the dehydroxylation of Mg(OH)_2_ or loss of bound water from the surface. The TGA of pristine PPEG (Figure 3C) indicates that the polymer completely decomposed at 400°C in accordance with the typical degradation temperature for PEG‐based polymers. For the one‐pot and post‐functionalized samples (Figure 3D, E), the mass loss contained mainly three decomposition windows, namely 100°C–250°C, 250°C–420°C, and 420°C–600°C. The mass loss between 100°C and 250°C indicates the presence of adsorbed water or a hydroxide layer. The decomposition step observed around 250°C–420°C, which is also present for pristine PPEG, confirms the presence of the polymer in both samples. The polymer loadings were determined to be 18 wt% for the one‐pot sample and 4 wt% for the post‐functionalized sample. A slight mass increase observed for the post‐functionalized material could be attributed to the formation of metal oxides or to moisture and gas uptake, including CO_2_, which may convert to MgCO_3_ and decompose above 500°C. The one‐pot and post‐functionalized samples retained 70% and 91% of their mass, respectively, reflecting the high decomposition temperature of the Mg core. Taken together, the FTIR and TGA data confirm the presence of PPEG on the surface of the MgNPs.

To test the dispersibility and structural stability toward oxidation in water, the original dispersant (propan‐2‐ol) was removed upon centrifugation and replaced with the same volume of milli‐Q water. The color change was subsequently monitored by exploiting the characteristic black appearance of bare MgNPs, arising from the strong plasmonic absorption of the metallic core. Upon exposure to ultrapure water, the black coloration progressively transformed into the white hue of Mg(OH)_2_, providing a straightforward visual indicator of surface oxidation. All samples generated gas bubbles accompanied by the formation of white precipitates within 5 min, reflecting the strong tendency of metallic Mg to undergo rapid oxidation upon contact with water. The pre‐added PPEG samples (method 3) showed almost no protection toward water, similar to the behavior of bare MgNPs, indicating inefficient binding of the phosphonate ion onto the surface. The one‐pot (method 2) and post‐functionalized samples (method 1) exhibited water stability for at least 40 min and 3 h, respectively. The one‐pot synthesis route with PPEG leads to a nanostar‐like morphology, while the post‐functionalization routes result in nanoplatelets. As confirmed by TGA results, nanostars contain a higher amount of PPEG than the nanoplatelets. Since the nanostars are expected to have a larger surface area than the nanoplatelets due to their morphology and size, this implies that the density of PPEG/nanostar is lower than PPEG/nanoplatelet, making the nanostars much more accessible to water and oxidation. To distinguish sample coloration from background effects, the color‐intensity analysis (Table S2 and Figure 4) was performed using ImageJ 1.54 g by calculating the ratio between the mean intensity of a defined region (*A* = 1518 pixels) in the sample and that of the background. This method reduced the signal‐to‐noise ratio associated with illumination variability. Ratios near zero indicated that the sample preserved the characteristic black color of metallic MgNPs, whereas ratios approaching or exceeding unity reflected the transition toward the white Mg(OH)_2_ phase. Overall, this work provides a promising strategy for optimizing colloidal stability in both the star‐like one‐pot sample and the post‐functionalized hexagonal platelet sample. Future improvements may involve tuning the polymer chain length and concentration to enhance steric stabilization, thereby improving dispersion efficiency and preserving MgNP plasmonic properties for water‐based applications such as biomedical environments.

## 3 Conclusion

In this work, we have demonstrated that α‐methoxy‐ω‐phosphonic acid poly(ethylene glycol) (PPEG1000) is an effective ligand for stabilizing colloidally synthesized magnesium nanoparticles toward oxidation in water. By systematically varying the timing of PPEG addition, we revealed its decisive influence on nucleation, growth, and the resulting nanoparticle morphology. Post‐functionalization preserved the intrinsic hexagonal platelet structure, whereas one‐pot introduction after initial nucleation arrested growth and produced star‐like nanoclusters. In contrast, PPEG pre‐addition disrupted Mg(II) reduction, yielding poorly defined polymer‐metal aggregates. FTIR and TGA confirmed phosphonate binding, and water‐dispersion assays showed enhanced resistance of functionalized MgNPs to oxidation, extending their lifetime from minutes to hours. These findings highlight phosphonic‐acid PEG ligands as promising passivation agents and underscore functionalization timing as a powerful parameter for controlling the stability and morphology of reactive metallic magnesium nanoparticles.

## Conflicts of Interest

The authors declare no conflicts of interest.

## Supporting information




**Supporting File**: chem71008‐sup‐0001‐SuppMat.docx.

## Data Availability

Data available on request from the authors.
