# Supplementary material for: Enhancing Stability of Metallic Magnesium Nanoparticles toward Oxidation in Water via PEG‐Phosphonate Passivation
Source: Chemistry. 2026 Apr 11;32(24):e03619. doi: 10.1002/chem.202503619 (PMC13290416; doi:10.1002/chem.202503619)

Supporting Information

Enhancing Stability of Metallic Magnesium Nanoparticles Toward Oxidation in Water via PEG-Phosphonate Passivation

Anupong Nuekaew,^[a]^ Delphine Talbot ^[a]^ and Ali Abou-Hassan*^[a],[b]^

[a] A. Nuekaew, D. Talbot, Prof. A. Abou-Hassan
PHysicochimie des Électrolytes et Nanosystèmes InterfaciauX (PHENIX)
Sorbonne Université, CNRS, 75005 Paris, France

[b] Prof. A. Abou-Hassan
Institut Universitaire de France (IUF), 75231 Paris, France
Email: ali.abou_hassan@sorbonne-universite.fr

Materials and Methods

Naphthalene, Li pellets and Di-n-butylmagnesium (0.5 M in hexane), anhydrous isopropanol and anhydrous tetrahydrofuran were purchased from Sigma Aldrich. All chemicals used for the syntheses are of analytical purity grade and they are used without further purification. All glassware was washed with ethanol (96%), acetone (>99 %) and agua regia containing hydrochloric acid (HCl, 37%) and nitric acid (HNO_3_, 68%).

Synthesis of MgNPs

Magnesium nanoparticles were synthesized using a published procedure as described in reference 14 of the manuscript. Naphthalene (350 mg, 2.73 mmol), and Li pellets (60 mg, 8.65 mmol) were sonicated for 25 min in THF (20 mL) under N_2_ atmosphere, obtaining the dark green solution. Di-n-butylmagnesium (3 mL, 0.5 M) was then added to the obtained dark green solution of lithium napthalenide at 53 °C and 300 rpm under N_2_ atmosphere. After 1h, the reactive LiNapht was then quenched using propan-2-ol (20 mL). The crude particle was then washed with tetrahydrofuran (2 x 20 mL) and propan-2-ol (2 x 20 mL), respectively. The final particles were redispersed in propan-2-ol (20 mL).

**Functionalization of MgNPs with PPEG1000**

Three MgNP@PPEG samples were prepared using identical reagents and workup conditions but with different sequences of PPEG1000 addition. In the post-functionalization route, a PPEG1000 solution in THF was added to the freshly synthesized MgNP dispersed in THF under sonication, followed by 2h sonication and overnight incubation at room temperature. The particles were then washed with IPA (3 x 20 mL) and redispersed in IPA (20 mL). In the one-pot route, naphthalene and Li were first sonicated in THF under N_2_ before adding di-n-butylmagnesium. After 5 min, the PPEG1000 solution was injected, stirred for 1h, quenched with IPA, washed with THF and IPA, and redispersed in IPA. In the pre-addition of PPEG in LiNapht route, the same one-pot procedure was followed except that the PPEG1000 solution was added to lithium naphthalenide before introducing di-n-butylmagnesium with identical quenching, washing and redispersion steps.

The solution of PPEG1000 (100 mg) in tetrahydrofuran (20 mL) was added to the as-prepared magnesium nanoparticle redispersed in THF (20 mL) while sonicating. The mixture was sonicated for 2 h and incubated at room temperature overnight. The particles were then washed with propan-2-ol (3 x 20 mL) and redispersed in propan-2-ol (20 mL).

**Characterization**

TEM measurements were carried out using a JEOL-1011 transmission electron microscope operating at 100 kV. Before analysis, each corresponding sample was deposited on a holey carbon copper grid by casting a drop of sample in IPA solution, followed by the evaporation of the solvent under room conditions overnight.

The UV-Vis-NIR spectra (300-1000 nm) were recorded at the room temperature in a 1 cm quartz cuvette using an Avantes spectrophotometric setup composed of an AvaLight-DHc lamp connected by optical fibers to a StarLine AvaSpec UV/Vis detector and to a NIRLine AvaSpec-NIR256-1.7 NIR detector.

IR spectra of powder of samples were acquired with a Thermo Nicolet iZ10 FTIR (Thermo Scientific Instrument) spectrophotometer with a resolution of 4 cm^-1^ between 400 and 4000 cm^-1^ in ATR mode for 128 scans on a diamond crystal in air. The nanoparticle dispersion was dried to the powder form at 70 °C overnight.

Thermogravimetric analysis was performed with a TGA 550 (Thermal Analysis Instrument). The samples were heated from room temperature to 620 °C at a rate of 10 °C/min in the TGA instrument under nitrogen gas at a flow rate of 50 mL/min.

For water dispersion assay, each sample in propan-2-ol (375 µL) was centrifuged at 5000 rpm, 5 min at room temperature. The solvent was replaced with ultrapure water, pipette mix, briefly sonicated and allowed for visual observation.

**Figure S1.** Color of (A) bare MgNP, (B) MgNP-PPEG obtained by post-functionalization (method 1), (C) MgNP-PPEG obtained by one-pot functionalization (method 2), and (D) MgNP-PPEG synthesized in the presence of PPEG pre-added in LiNapht (method 3).


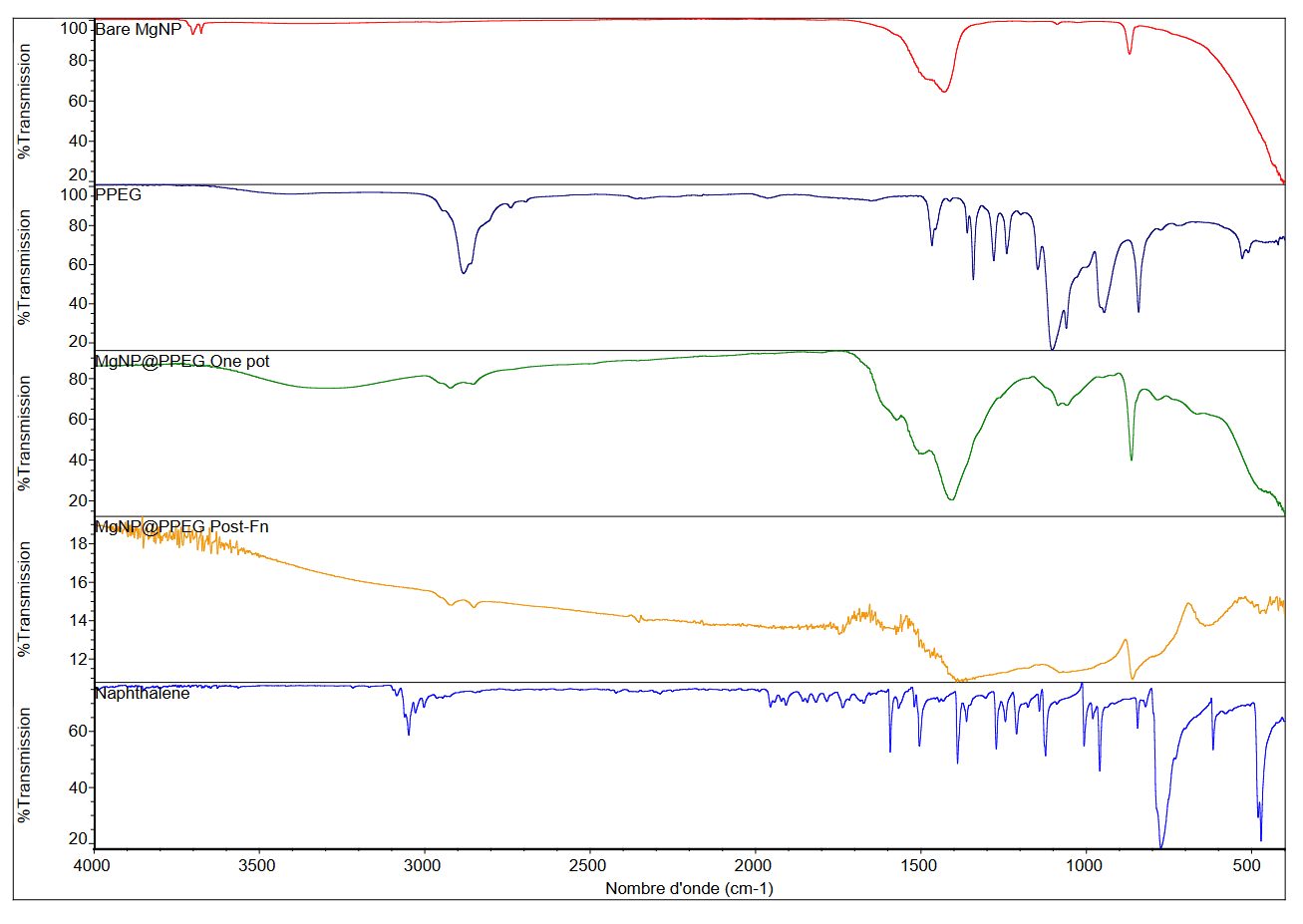


**Figure S2.** FTIR spectra of bare MgNPs, pristine PPEG, MgNP-PPEG obtained by one-pot synthesis (method 2), MgNP-PPEG obtained by post-functionalization (method 1), in comparison to pristine naphthalene, showing the absence of impurities.

**Table S1.** Assignment of the main FTIR bands observed for bare MgNPs, pristine PPEG, and PPEG-functionalized MgNPs.^[15-18]^

| **Wavenumber (cm^-1^)** | **Assignment** | **Origin** |
| --- | --- | --- |
| ~2922 | C-H asymmetric stretching | PEG backbone (-CH_2_-) |
| ~2851 | C-H symmetric stretching | PEG backbone |
| ~1574-1496 | CH_2_ bending | PEG backbone |
| 1300-1000 | P-O / P=O stretching | Phosphonate group |
| ~950-900 | P-O-H vibration | Phosphonic acid (pristine PPEG) |
| ~850 | Mg-O stretching | Surface MgO layer |
| ~783 | C-H out-of-plane vibration | PEG/phosphonate structure |
| ~640 | Skeletal vibration | Polymer structure |

**Table S2.** Time-dependent color change of MgNPs in IPA (375 µL) redispersed in water (375 µL) after centrifugation for different functionalization protocols: bare MgNPs, pre-added PPEG in LiNapht, one-pot MgNP-PPEG, and post-functionalized MgNP-PPEG. Representative images of each sample at selected time points are shown, with the corresponding color intensity ratio values indicated near each image. The ratio was calculated by dividing the color intensity of a selected area by the intensity of the background, illustrating the time-dependent stability and color evolution of each sample. These values were used to generate the colorimetric ratio vs. time graph in Figure 3C.


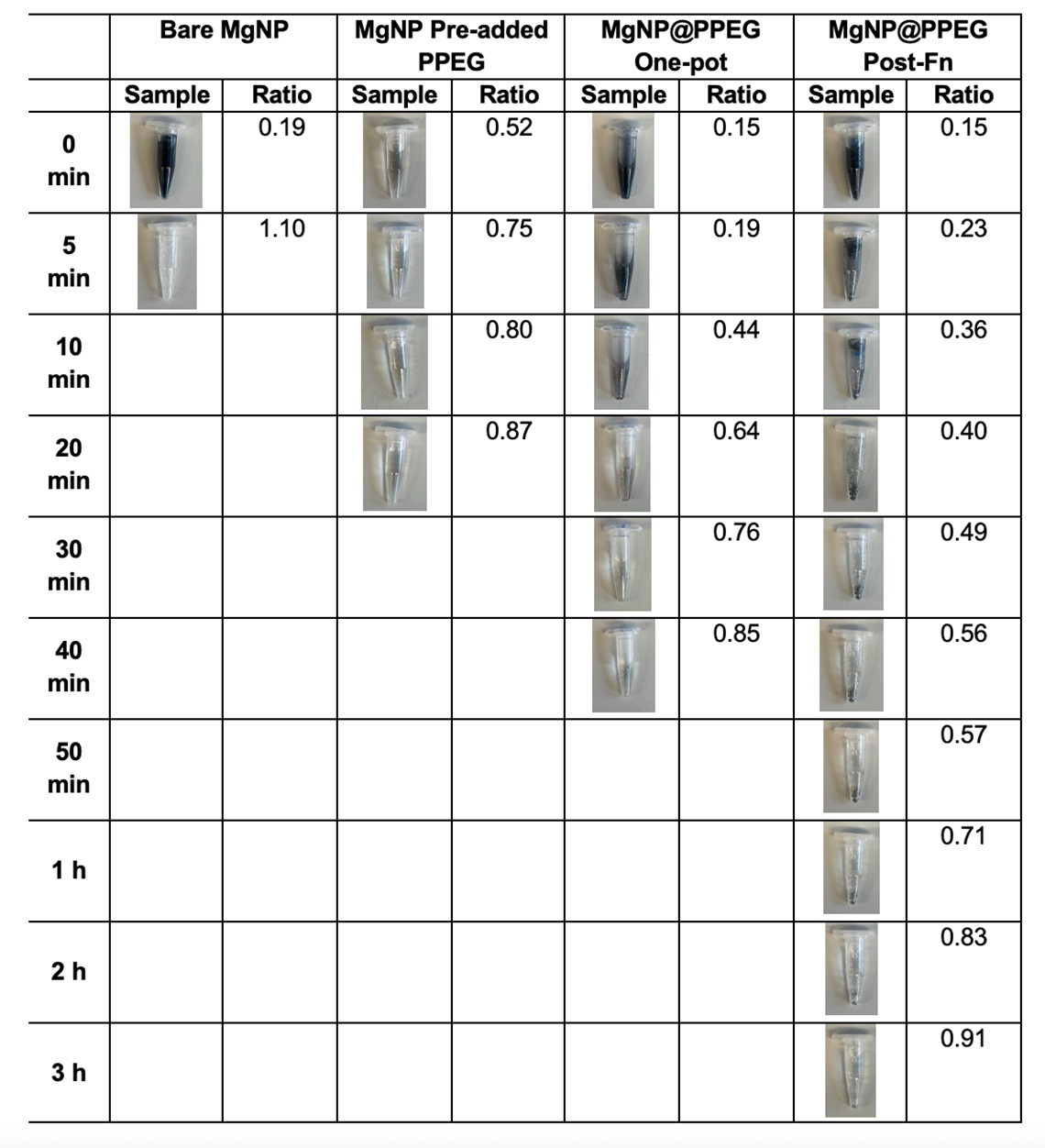

Supplement: Supplementary file 1 — Supporting File: chem71008‐sup‐0001‐SuppMat.docx. [file CHEM-32-e03619-s001.docx]
